# Potential diagnostic markers and therapeutic targets for obstructive sleep apnea with comorbid depression based on bioinformatics analysis

**DOI:** 10.3389/fgene.2025.1655000

**Published:** 2025-11-19

**Authors:** Yinfei Lu, Zao Tang, Xiangyu Zhou, Wanting Lin, Xiao Guo

**Affiliations:** 1 Department of Geriatrics, WuHan Red Cross Hospital, Wuhan, Hubei Province, China; 2 Department of Orthopedics, Wuhan Fourth Hospital, Wuhan, Hubei Province, China; 3 Minda Hospital, Hubei Minzu University, EnShi, Hubei Province, China; 4 Department of Ophthalmology, Union Hospital, Tongji Medical College, Huazhong University of Science and Technology, Wuhan, Hubei Province, China; 5 Department of Urology, Union Hospital, Tongji Medical College, Huazhong University of Science and Technology, Wuhan, Hubei Province, China

**Keywords:** obstructive sleep apnea, major depressive disorder, hub gene, WGCNA, bioinformatics analysis

## Abstract

**Background:**

Obstructive sleep apnea (OSA) and major depressive disorder (MDD) impose substantial quality-of-life burdens and socioeconomic costs. Growing evidence indicates bidirectional disease interactions that exacerbate clinical outcomes. This study identifies diagnostic biomarkers and explores therapeutic targets underlying OSA-MDD comorbidity.

**Methods:**

We analyzed OSA/MDD-specific differentially expressed genes (DEGs) from Gene Expression Omnibus (GEO) datasets. Weighted gene co-expression network analysis (WGCNA) identified co-expressed modules. Protein-protein interaction (PPI) networks derived key genes via STRING. Diagnostic markers were established through dual-algorithm screening, with immune associations and therapeutic potential assessed. Finally, *in vitro* validation confirmed key findings.

**Results:**

We identified 77 comorbid OSA-MDD DEGs. Integrated WGCNA-PPI analysis revealed eight key hub genes. LASSO regression nominated three diagnostic markers, including CD74 (CD74 molecule), RPL26L1 (ribosomal protein L26 like 1), and MRPL9 (mitochondrial ribosomal protein L9). MRPL9 was excluded for low diagnostic value for OSA and MDD. CD74 and RPL26L1 markers correlated with immune cell infiltration in OSA and MDD. *In vitro*, intermittent hypoxia significantly upregulated CD74 and RPL26L1 in microglia versus normoxia controls.

**Conclusion:**

CD74 and RPL26L1 represent mechanistically grounded diagnostic biomarkers and therapeutic targets for OSA-MDD comorbidity. Shared pathways offer novel intervention opportunities for both conditions.

## 1 Introduction

Obstructive sleep apnea (OSA), a prevalent chronic sleep-related breathing disorder, involves recurrent partial or complete upper airway collapse during sleep and manifests as excessive daytime sleepiness, fatigue, and recurrent breathing interruptions (apneas, snoring, and gasping). This pathophysiology triggers sleep fragmentation, intermittent hypoxia (IH), sympathetic overactivity, and homeostatic disruption (Gottlieb and Punjabi, 2020). Epidemiologically, OSA affects 9%–38% of the general population, with males disproportionately affected (13%–33%) compared to females (6%–19%) (Senaratna et al., 2017). Strong clinical evidence associates OSA with elevated risks of mental, metabolic and cardiovascular comorbidities, including major depressive disorder (MDD), coronary heart disease, type 2 diabetes, hypertension, and stroke (Drager et al., 2017; Reddy et al., 2022). Elucidating OSA’s underlying mechanisms and risk factors is therefore essential for developing targeted preventive and therapeutic interventions.

MDD, characterized by persistent alterations in mood, cognition, and physical functioning, significantly increases functional disability and mortality. Affecting 22.1% of the global population, it represents a major public health burden. Growing evidence indicates a bidirectional relationship between MDD and OSA, with comorbid prevalence rates of ∼18% (MDD with OSA) and ∼17.6% (OSA with MDD) (Shakir et al., 2011). Shared pathophysiological mechanisms likely contributing to this comorbidity include: 1) Hypoxemia-mediated neuropathology: Sleep fragmentation and white matter changes exacerbate MDD symptoms (Kerner and Roose, 2016). 2) Inflammatory dysregulation: OSA-induced hypoxia elevates MDD-associated cytokines (interleukin-6 and tumor necrosis factor-α) (Gupta and Simpson, 2015). 3) Serotonergic dysfunction: Shared pathways in airway control and mood regulation (Cheng, 2018). 4) Common comorbidities: Obesity, cardiovascular disease, hypertension, and diabetes amplify symptom burden (Schröder and O'Hara, 2005). We postulate that OSA potentiates MDD severity through these biological pathways and hypothesize shared genetic mechanisms underlie OSA-MDD comorbidity. Elucidating these convergent pathological processes is essential for identifying novel therapeutic targets.

Multiple studies have shown that hypoxia exposure induces the expression of susceptibility genes and epigenetic reprogramming which includes altering histone modifications, noncoding RNA expression, and DNA methylation patterns (Xia et al., 2009; Tausendschön et al., 2011; Shmakova et al., 2014; Cortese et al., 2017; Nanduri et al., 2017; Barreca et al., 2021; Zhou TT et al., 2023). Despite these established relationships, no multi-omics studies have investigated the epigenetic and genomic landscape in comorbid OSA and MDD. Therefore, comprehensive profiling of these interactions warrants investigation to elucidate shared pathophysiological mechanisms.

**FIGURE 1:**
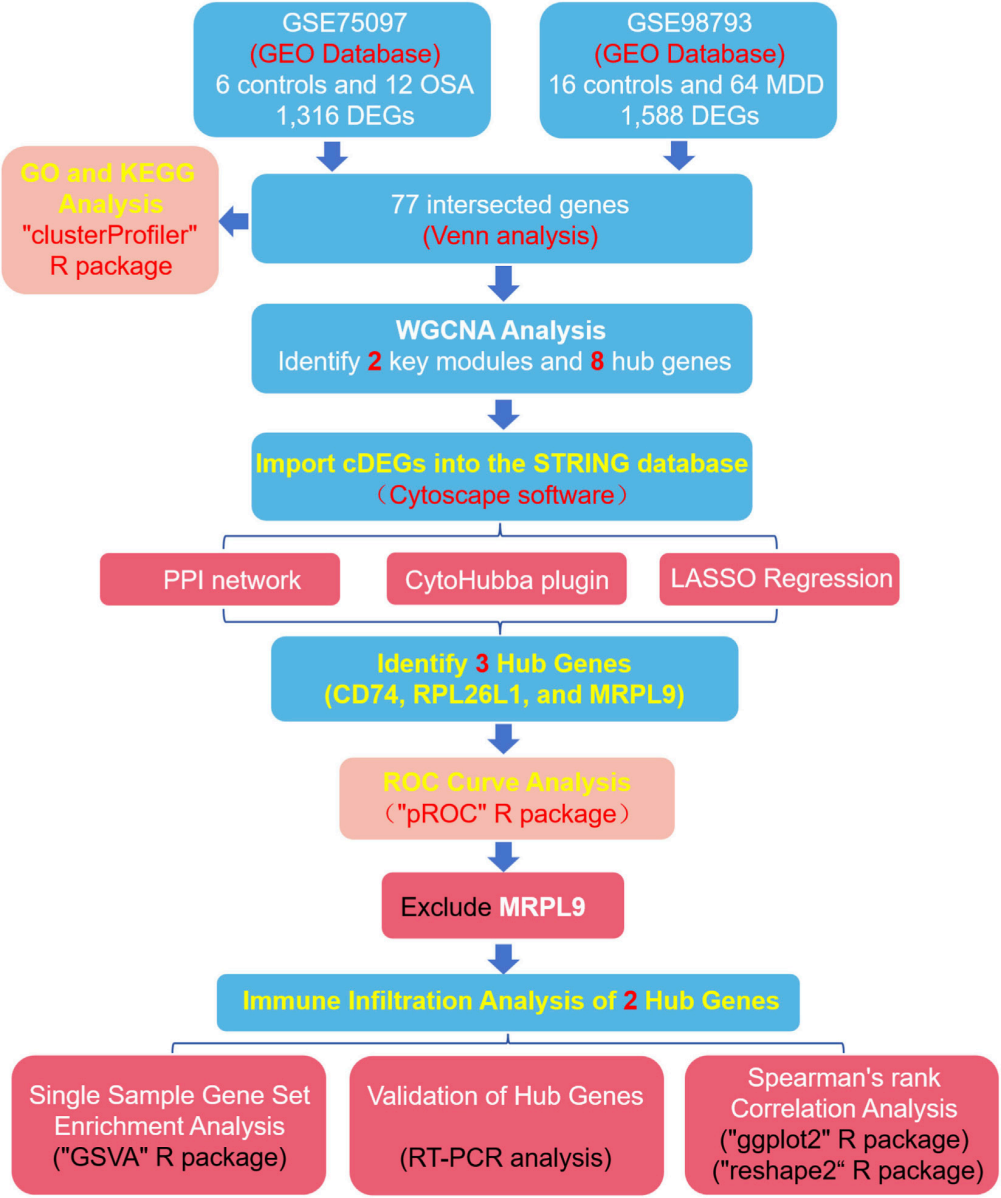
The workflow diagram of the study.

This study investigates shared genetic mechanisms between OSA and MDD to elucidate the underlying biology of their comorbidity. We identified diagnostic biomarkers from these common genes and evaluated their association with dysregulation in mitochondrial, oxidative phosphorylation (OXPHOS), and antigen presentation pathways, immune cell infiltration, therapeutic potential, and diagnostic utility. The workflow diagram of this study was displayed in Figure 1.

## 2 Materials and methods

### 2.1 Data collection and processing

The datasets used in this study were obtained from the Gene Expression Omnibus (GEO) database. The OSA group was selected from the GSE75097 dataset, which included peripheral blood mononuclear cells (PBMCs) from 12 patients with OSA and six healthy controls. Gene expression profiling was performed using the GPL10904 platform, Illumina HumanHT-12 V4.0 expression beadchip (gene symbol). The MDD group was selected from the GSE98793 dataset, which included whole blood samples from 64 patients with MDD and 16 healthy controls, profiled on the GPL570 platform ([HG-U133_Plus_2] Affymetrix Human Genome U133 Plus 2.0 Array). Another MDD group was selected from the GSE19738 dataset, which included whole blood samples from 34 patients with MDD and 33 healthy controls. To mitigate biases arising from combining samples processed in different batches, batch effect correction of the merged datasets was conducted using the ComBat method implemented in the sva package (v3.36.0) for R software (v4.0.2).

### 2.2 Differential gene expression analysis

We performed normalization, transformation, and differential expression analysis on RNA-Seq data using the DESeq2 package (v1.42.2) in R (v4.0.2). The dataset comprised samples from two groups: OSA and MDD. During data preprocessing, raw count data were first normalized. A DESeqDataSet was constructed using the DESeqDataSetFromMatrix function, followed by data transformation via the variance-stabilizing transformation (vst) or regularized logarithm (rlog) methods. To address batch effects, we applied batch effect correction using the SVA package (v3.36.0). Differential expression analysis was then conducted using the DESeq () function in DESeq2. Significantly differentially expressed genes (DEGs) were identified by applying stringent thresholds (|log_2_FC| > 1.0 and adjusted *P*-value <0.05). The overlap of DEGs between MDD and OSA was visualized using a Venn diagram generated with the VennDiagram package (v1.6.20). Finally, expression patterns of the DEGs were illustrated in a heatmap created with the pheatmap package (v1.0.12), where hierarchical clustering was applied to group samples based on similarity.

### 2.3 Weighted gene Co-expression network analysis (WGCNA)

In this study, WGCNA analysis was performed on the GSE98793 and GSE75097 datasets. Gene expression data were preprocessed using the WGCNA package (v1.70) in R, including data cleaning and normalization. Genes were clustered hierarchically, and modules were identified via the dynamic tree-cutting algorithm. A gene co-expression network was then constructed, and modules were partitioned using the blockwiseModules function in WGCNA. Network heatmaps were generated to visualize correlations among selected genes. To investigate relationships between gene modules and clinical traits, we calculated Pearson correlations between module eigengenes (MEs) and clinical features, visualized in a module-trait relationship heatmap. Finally, scatter plots of module membership (MM) versus gene significance (GS) were generated for each module. The correlation between MM and GS was computed to evaluate the biological relevance of modules to clinical traits.

### 2.4 Protein-protein interaction (PPI) network analysis

To identify candidate hub genes, we first performed intersection analysis on two significantly correlated modules from the WGCNA results (turquoise and black modules) to extract common genes. These overlapping genes were used to construct a PPI network using the STRING database (v11.0; https://string-db.org/). The network was visualized and analyzed in Cytoscape (v3.9.1). Within Cytoscape, we employed the cytoHubba plugin to perform topological analysis of network nodes. Hub genes were screened using multiple centrality metrics, including Degree, Betweenness, Closeness, and Eccentricity. Final hub genes were identified through consensus across these topological algorithms.

### 2.5 Functional enrichment analysis of hub genes

Functional enrichment analysis was performed on hub genes identified through WGCNA analysis using the ClusterProfiler package (v4.0.5). Gene Ontology (GO) enrichment analysis was conducted across all three domains: cellular component (CC), biological process (BP), and molecular function (MF). Pathway enrichment analysis was subsequently carried out using the Kyoto Encyclopedia of Genes and Genomes (KEGG) database to identify biological pathways associated with these genes. A significance threshold of adjusted *P*-value <0.05 was applied to all enrichment analyses. Results were visualized using dot plots, where the dot size represents the number of enriched genes and the color gradient indicates the significance level based on the adjusted *P*-value.

### 2.6 LASSO regression for diagnostic biomarker identification

LASSO (Least Absolute Shrinkage and Selection Operator) regression was employed for feature selection, applying L1 regularization to shrink coefficients and identify core genes associated with OSA and MDD. Support Vector Machine (SVM) implementation utilized the caret package (v6.0–86) with dependencies on kernlab (v0.9–29) and e1071 (v1.7–9). During model training, 10-fold cross-validation was applied to evaluate accuracy and misclassification error. This validated model performance and ensured generalization capability on independent datasets. The cross-validation procedure involved:

1. Random partitioning of data into 10 subsets.
2. Iterative training on 9 subsets and testing on the held-out subset.
3. Calculation of final accuracy metrics from average error rates across all folds.

We applied this machine learning pipeline separately to OSA and MDD gene expression datasets to extract disease-specific diagnostic biomarkers. Overlapping biomarkers between OSA and MDD represent shared diagnostic signatures for both disorders.

### 2.7 Immune infiltration analysis

Immune cell composition was evaluated using the CIBERSORT method to estimate relative abundances of immune cell types across samples. Single-sample gene set enrichment analysis (ssGSEA) was performed via the GSVA package (v1.44.0) in R to quantify enrichment levels of immune cell populations in individual samples. ssGSEA calculates enrichment scores by measuring the association between each sample’s gene expression profile and immune cell-specific gene signatures. Enrichment scores generated by GSVA were used for between-group comparisons of immune infiltration patterns. Differences in immune cell infiltration across experimental groups were statistically evaluated. Spearman’s rank correlation analysis was then applied to assess relationships between immune cell infiltration scores and expression levels of diagnostic biomarker genes. Results were visualized using scatter plots (for individual correlations) or heatmaps (for matrix-level correlations).

### 2.8 Murine BV-2 microglial cell culture and IH treatment

The murine BV-2 microglial cell line (Chinese Academy of Medical Sciences, Beijing) was cultured in Dulbecco’s Modified Eagle Medium (DMEM) supplemented with 10% fetal bovine serum and 1% penicillin/streptomycin (100 U/ml penicillin and 100 mg/mL streptomycin) at 37 °C under 5% CO_2_. Cells underwent IH in a custom chamber with oxygen cycling between 0% and 22% every 30 min for 48 h prior to real-time polymerase chain reaction (RT-PCR) analysis (Wu et al., 2021).

### 2.9 RT-PCR validation of hub genes

Total RNA was extracted from microglial cells (Hu et al., 2024), and cDNA synthesized using the PrimeScript RT Kit (Takara, #RR047A). Gene expression was quantified by qPCR with SYBR® Premix Ex Taq II (Takara, #RR430B), normalized to *gapdh*. Primer sequences used in the study were as follows:

CD73: Forward (5′-3′): AGT​GCG​ACG​AGA​ACG​GTA​AC; Reverse (5′-3′): CGT​TGG​GGA​ACA​CAC​ACC​A.

RPL26L1: Forward (5′-3′): TTC​AAT​CCC​TTC​GTT​ACC​TCG​G; Reverse (5′-3′): TAG​TGT​CCT​CGA​ACT​ACC​TGG.

GAPDH: Forward (5′-3′): AGG​TCG​GTG​TGA​ACG​GAT​TTG; Reverse (5′-3′): TGT​AGA​CCA​TGT​AGT​TGA​GGT​CA.

### 2.10 Statistical analysis

Bioinformatics analyses were performed using R software (v4.4.1). RT-PCR data represent mean ± SEM (n = 4 independent experiments) and were analyzed in GraphPad Prism 8. The relative proportions of immune cells in each sample were evaluated using the CIBERSORT/ssGSEA algorithm. Intergroup differences were compared via two-tailed t-tests (or Wilcoxon tests), with the Benjamini-Hochberg method applied for multiple test correction to control the false discovery rate (FDR). Meanwhile, effect sizes (Cohen’s d) were calculated to quantify the actual magnitude of differences. A difference was considered statistically significant when FDR <0.05. Normality was verified by Shapiro-Wilk test, with between-group differences assessed using unpaired Student’s t-tests. *P < 0.05; **P < 0.01; ***P < 0.001.

## 3 Results

### 3.1 Data acquisition and processing

Human blood sample datasets, including GSE75097 and GSE98793, were obtained from the NCBI GEO database. Analysis revealed a linear distribution trend in the expression matrices of both datasets, suggesting minimal batch effects (Supplementary Figures S1A,B). To further ensure data quality, we performed Principal Component Analysis (PCA). This analysis demonstrated clear separation of all samples within the OSA and MDD datasets before (Supplementary Figure S1C,D) and after (Supplementary Figure S1E,F) batch effect correction, confirming high data reproducibility.

### 3.2 Identification of DEGs between OSA and control groups

**FIGURE 2:**
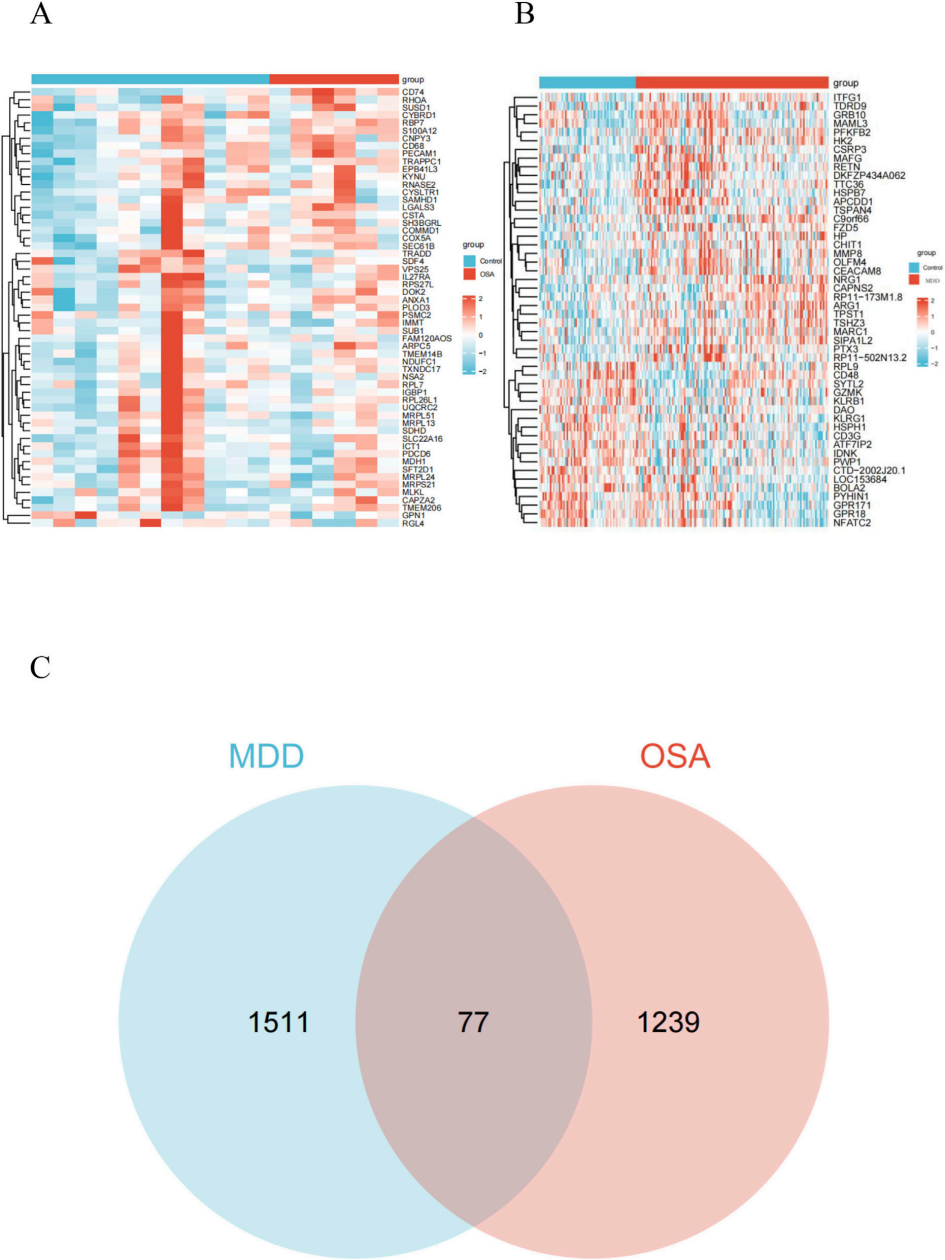
Identification of the key DEGs between OSA and MDD groups. **(A)** Heatmap showing the expression of genes in the OSA and control groups. **(B)** Heatmap showing the expression of genes in the MDD and control groups. **(C)** Venn diagram showing the overlap of genes between MDD and OSA groups.

We identified 1,588 differentially expressed genes (DEGs) in the GSE75097 dataset and 1,316 DEGs in the GSE98793 dataset. Key DEGs were selected using thresholds of adjusted *P*-value <0.05 and |log_2_FC| > 0. Heatmaps for both OSA and MDD datasets were shown in Figures 2A,B. Specifically, GSE75097 contained 654 upregulated and 662 downregulated genes. GSE98793 contained 864 upregulated and 724 downregulated genes. Venn analysis revealed 77 genes shared between the two datasets (Figure 2C).

### 3.3 Identification of co-expression gene modules

**FIGURE 3:**
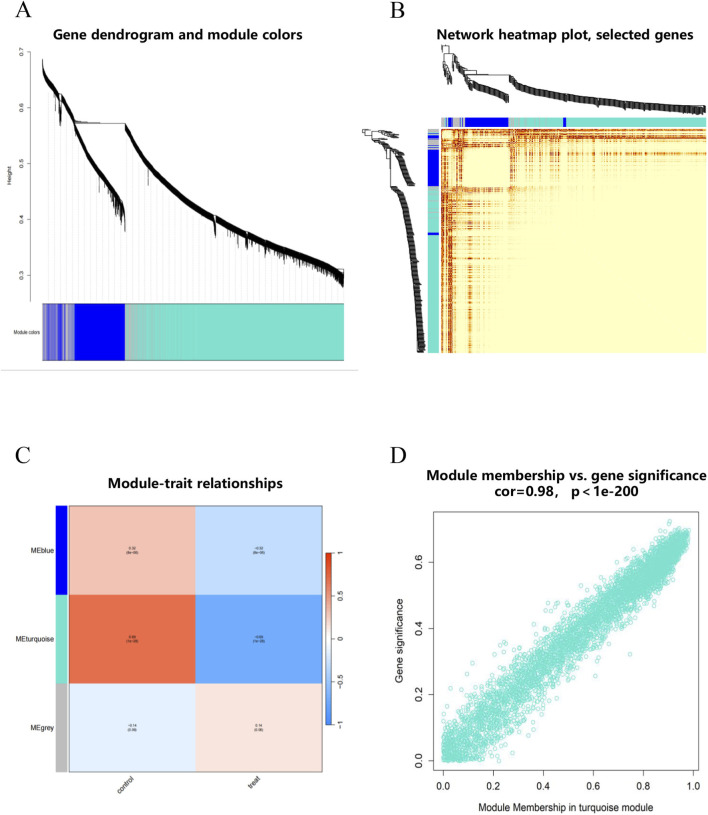
WGCNA analysis of GSE75097 dataset. **(A)** The gene dendrogram showed the hierarchical clustering of genes, with each color representing a different gene module identified by dynamic tree cutting. **(B)** Network heatmap of selected genes. **(C)** Module-trait relationships. The heatmap showed the correlation between each gene module and clinical traits. **(D)** Module membership vs. gene significance.

We performed WGCNA analysis on the GSE75097 dataset to identify co-expressed gene modules. Samples were stratified by disease status (OSA patients vs. healthy controls) with no outliers detected (Supplementary Figure S2A). For the OSA group, a soft-thresholding power (*β*) of 10 was selected to achieve scale-free topology (*R* ^2^ > 0.9) while maintaining high mean connectivity (Supplementary Figure S2B,C). Hierarchical clustering with dynamic branch cutting identified three co-expression modules (Figure 3A,B; Supplementary Figure S2D). To pinpoint OSA-relevant key modules, we calculated GS representing gene-trait correlations of three co-expression modules (MEblue, MEgrey, and MEturquoise modules), respectively, and found that MEturquoise module showed the highest GS (Figure 3C). Notably, within the MEturquoise module, GS exhibited a strong positive correlation with 3,372 module eigengenes (Figure 3D).

**FIGURE 4:**
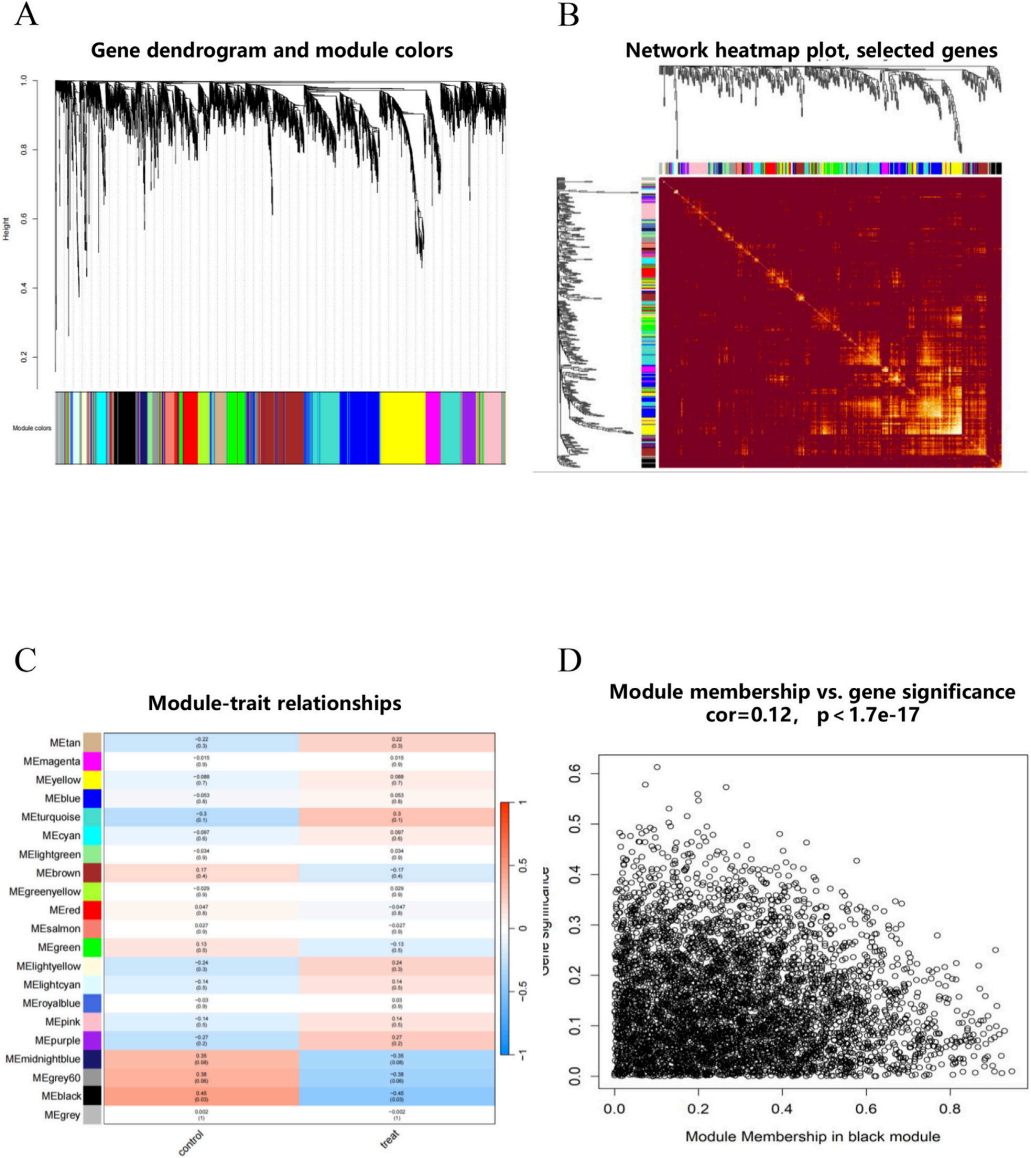
WGCNA analysis of GSE98793 Dataset. **(A)** The gene dendrogram showed the hierarchical clustering of genes, with each color representing a different gene module identified by dynamic tree cutting. **(B)** Network heatmap of selected genes. **(C)** Module-trait relationships. The heatmap showed the correlation between each gene module and clinical traits. **(D)** Module membership vs. gene significance.

Similarly, we performed WGCNA analysis on the GSE98793 dataset to identify co-expression modules. After stratifying samples by disease status (MDD patients vs. healthy controls) with no outliers detected (Supplementary Figure S3A), we selected a soft-thresholding power (*β* = 12) for the MDD cohort to achieve scale-free topology (*R* ^2^ > 0.9) while preserving high mean connectivity (Supplementary Figure S3B,C). Hierarchical clustering with dynamic branch cutting resolved 21 co-expression modules (MEtan, MEmagenta, MEyellow, MEblue, MEturquoise, MEcyan, MElightgreen, MEbrown, MEgreenyellow, MEred, MEsalmon, MEgreen, MElightyellow, MElightcyan, MEroyalblue, MEpink, MEpurple, MEmidnightblue, Megrey60, MEblack, and Megrey modules) (Figures 4A,B; Supplementary Figure S3D). GS calculation identified the MEblack module as having the highest association with MDD (Figure 4C). Within this module, GS exhibited a strong positive correlation with the expression of 248 module eigengenes (Figure 4D).

### 3.4 Identification of the key genes

**FIGURE 5:**
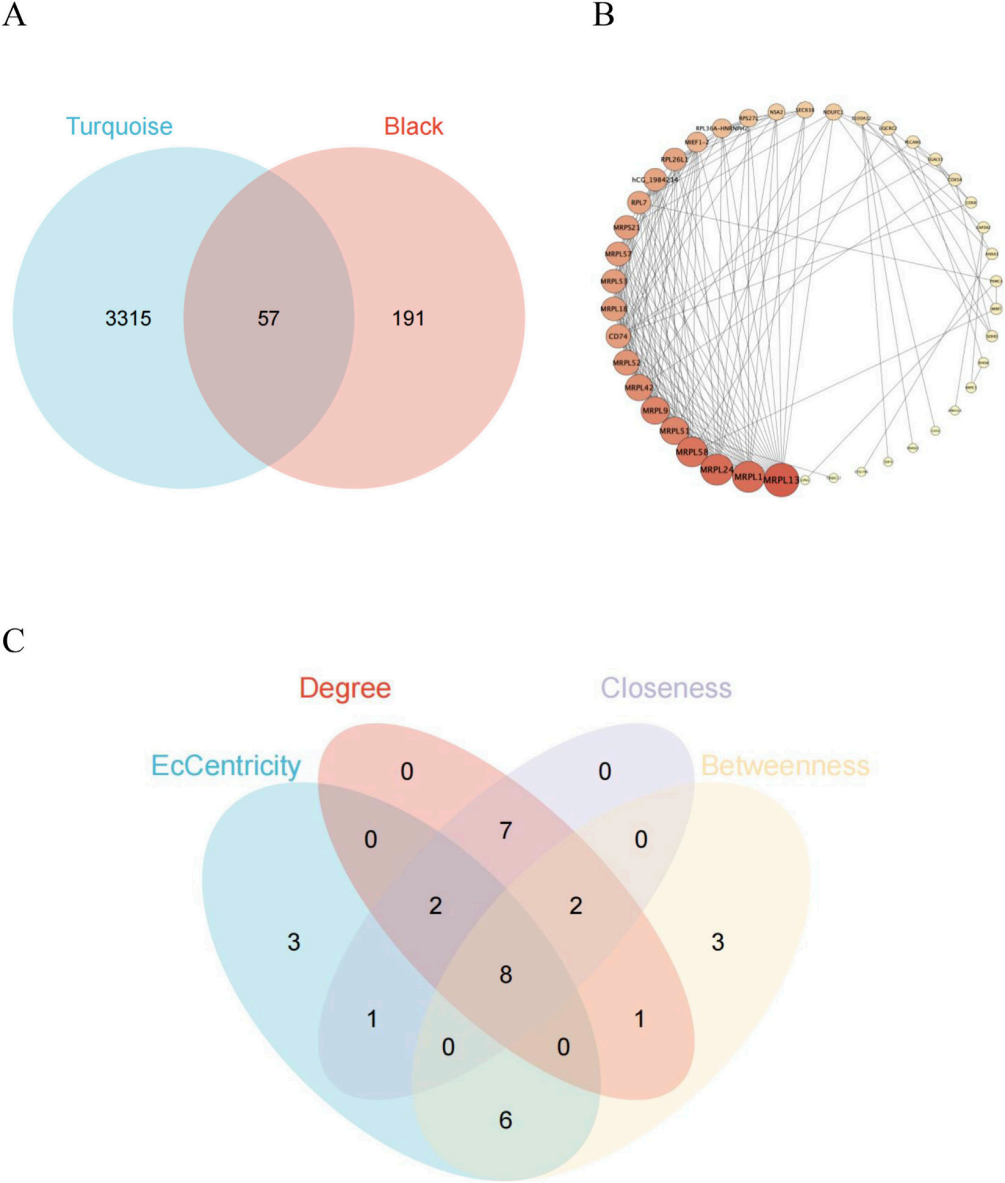
Based on the STRING database, PPI network analysis for the two diseases identified eight key genes. **(A)** Venn diagram of genes between the MEturquoise and MEblack modules. **(B)** Gene network plot of the MEblack module, showing the correlation and network structure between genes. The node color and edge thickness represented the importance of genes and their relationships. **(C)** Gene overlap under network centrality metrics. The Venn diagram showed the distribution of genes across four centrality metrics: Degree, EcCentricity, Closeness, and Betweenness.

In order to identify the key genes between OSA and MDD diseases, we intersected the 3,372 MEturquoise module eigengenes in OSA dataset and the 248 MEblack module eigengenes in MDD dataset, and obtained 57 intersected genes. Subsequently, interaction data for all 57 genes comprising the OSA-MEturquoise and MDD-MEblack co-expression modules were retrieved from the STRING database and visualized as PPI networks in Cytoscape software (Figures 5A,B). Using four topological features (Degree, Eccentricity, Closeness, and Betweenness), we filtered nodes with low connectivity (Degree <5). From the remaining nodes, the top 20 scorers for each feature were identified. The intersection of these top-scoring gene sets across all four features yielded eight hub genes (Figure 5C).

### 3.5 Functional enrichment analysis of hub genes

**FIGURE 6:**
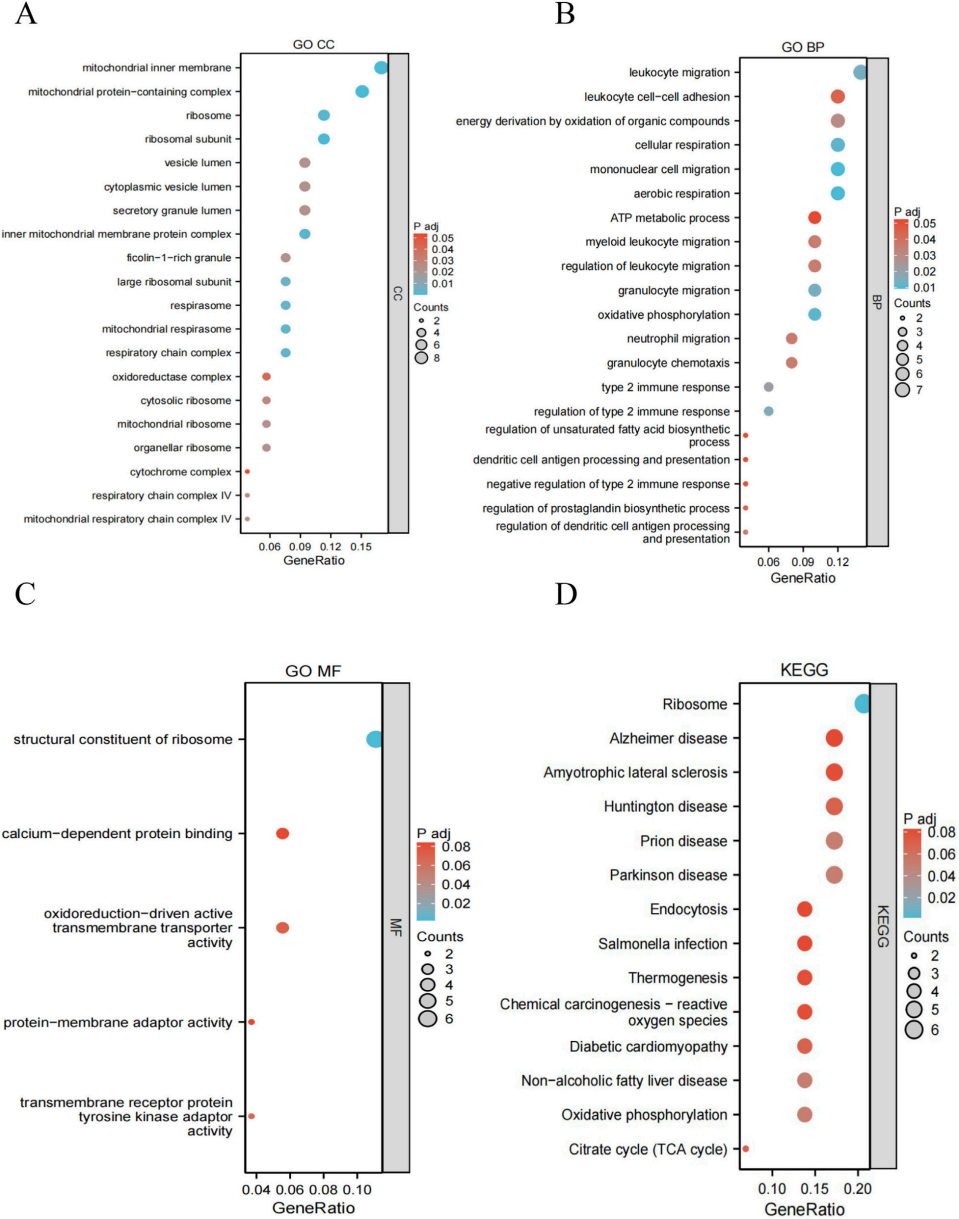
Functional enrichment analysis of eight key genes. **(A)** GO Cellular Component enrichment analysis. **(B)** GO Biological Process enrichment analysis. **(C)** GO Molecular Function enrichment analysis. **(D)** KEGG pathway enrichment analysis.

GO enrichment analysis of the eight DEGs shared between OSA and MDD was performed using the clusterProfiler R package (Figures 6A–C; Supplementary Table S1). This identified 95 significant GO terms: 58 Biological Processes (BP), five Molecular Functions (MF), and 32 Cellular Components (CC). Regarding CC, the hub genes were mainly enriched in the mitochondrial inner membrane (GO:0005743), mitochondrial protein-containing complex (GO:0098798), ribosome (GO:0005840), ribosome subunit (GO:0044391), vesicle lumen (GO:0031983), and cytoplasmic vesicle lumen (GO:0060205). As for BP, the hub genes were mainly enriched in the leukocyte migration (GO:0050900), leukocyte cell-cell adhesion (GO:0007159), energy derivation by oxidation of organic compounds (GO:0015980), cellular respiration (GO:0045333), mononuclear cell migration (GO:0071674), and aerobic respiration (GO:0009060). Finally, regarding MF, the hub genes were mainly enriched in the structural constituent of ribosome (GO:0003735), calcium-dependent protein binding (GO:0048306), oxidoreduction-driven active transmembrane transporter activity (GO:0015453), protein-membrane adaptor activity (GO:0043495), and transmembrane receptor protein tyrosine kinase adaptor activity (GO:0005068).

KEGG pathway analysis (Figure 6D; Supplementary Table S2) revealed enrichment in 14 pathways, mainly including Ribosome (hsa03010), Alzheimer’s disease (hsa05010), Amyotrophic lateral sclerosis (hsa05014), Huntington’s disease (hsa05016), Prion disease (hsa05020), Parkinson’s disease (hsa05012), Endocytosis (hsa04144), *Salmonella* infection (hsa05132), Thermogenesis (hsa04714), Chemical carcinogenesis-reactive oxygen species (hsa05208), Diabetic cardiomyopathy (hsa05415), Non-alcoholic fatty liver disease (hsa04932), Oxidative phosphorylation (hsa00190), and Citrate cycle (hsa00020). These shared enrichments suggested significant overlap in the molecular mechanisms underlying OSA and MDD.

### 3.6 The diagnostic values of hub genes

**FIGURE 7:**
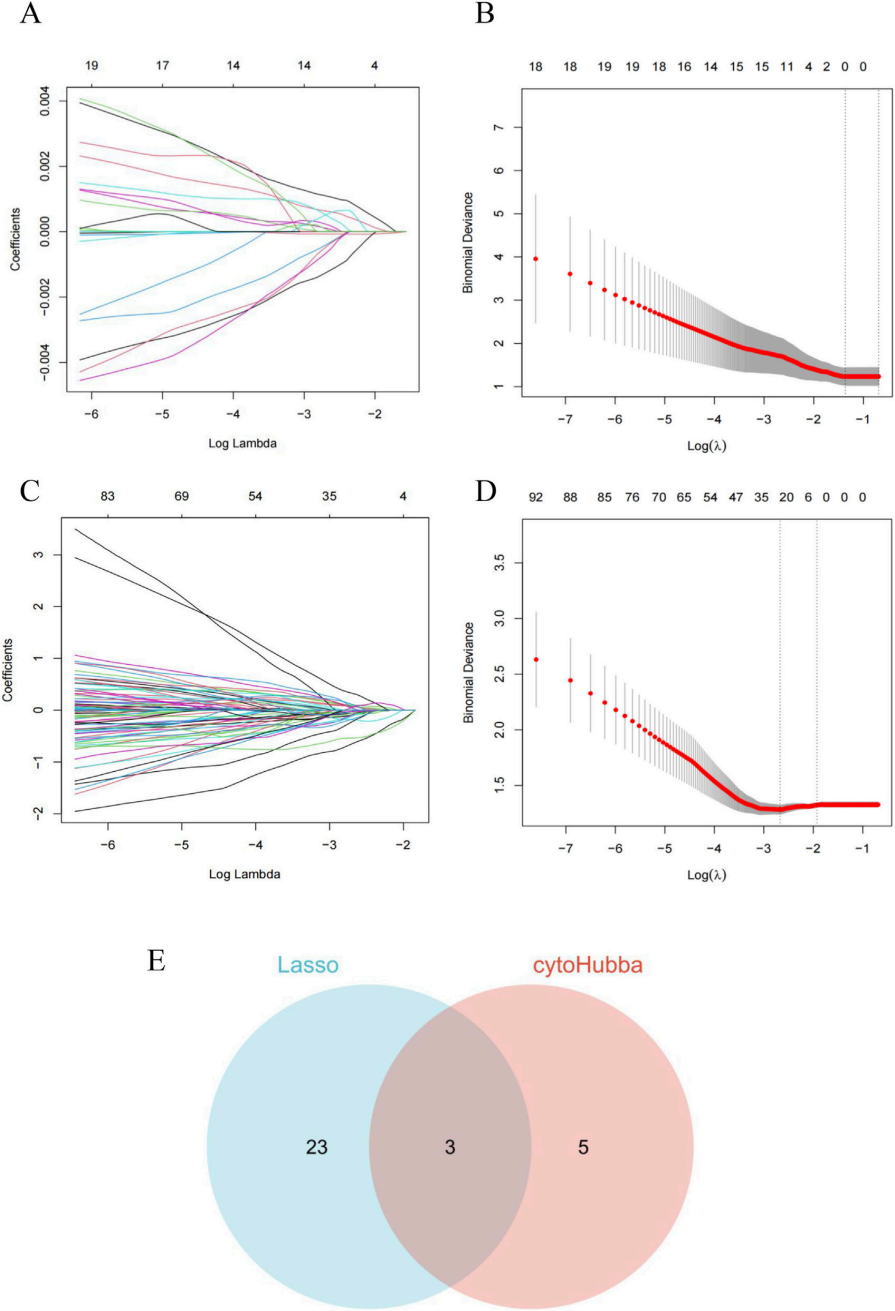
Core gene selection and validation based on machine learning methods. **(A,B)** Lasso regression path plots, showing the changes in the coefficients of each feature at different Lambda values. **(C,D)** CytoHubba analysis path plots, illustrating the selection process of key genes in the network. **(E)** Venn diagram showing the intersection of genes selected by Lasso regression and cytoHubba analysis, with three genes appearing in both methods.

**FIGURE 8:**
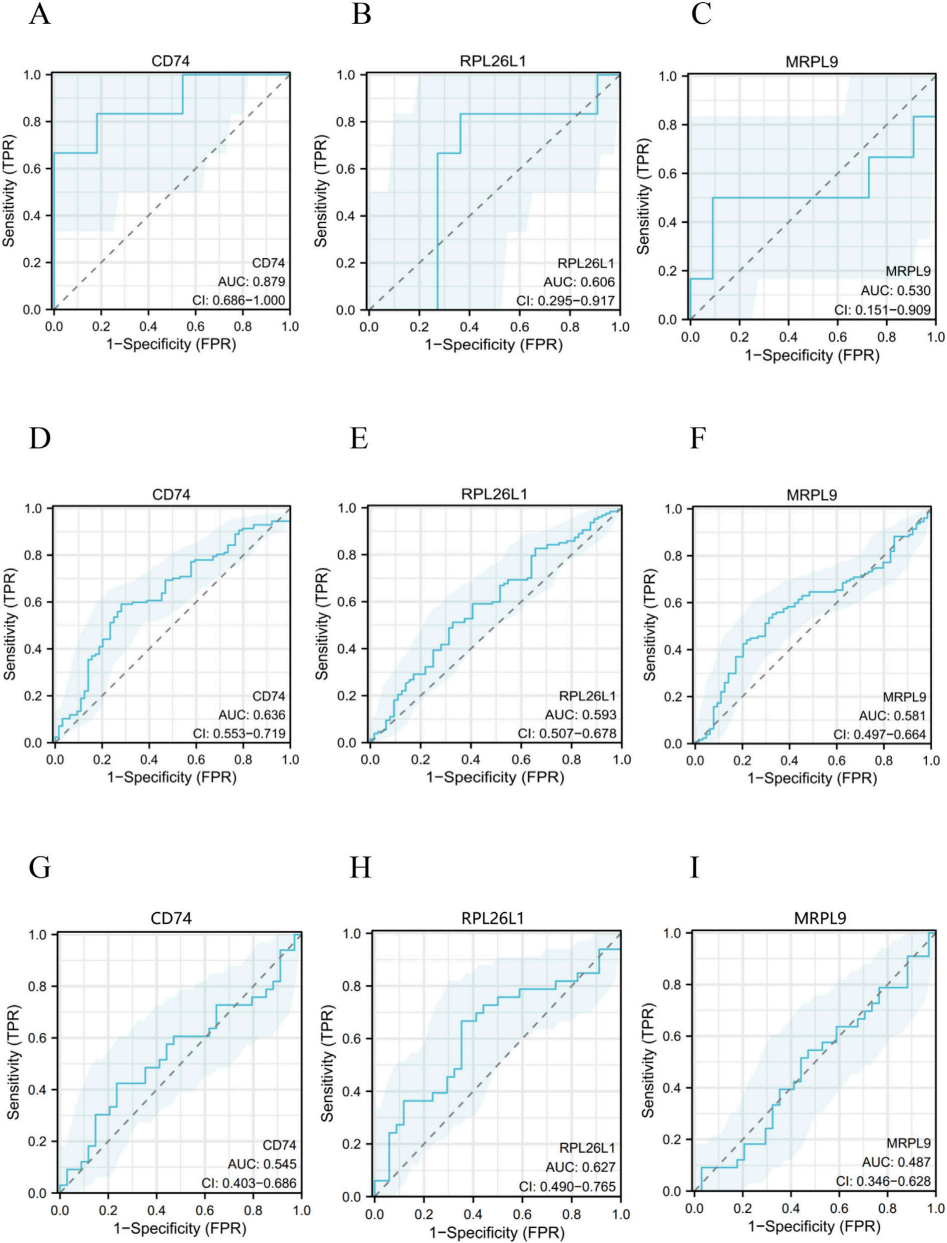
The ROC curves of the three common core genes in OSA and MDD. (**A–C**) ROC curves showing the diagnostic values of the key genes CD74 and RPL26L1, and MRPL9 in the GSE75097 (OSA) dataset. (**D–F**) ROC curves showing the diagnostic values of the key genes CD74, RPL26L1, and MRPL9 in the GSE98793 (MDD) dataset. (**G–I**) ROC curves showing the diagnostic values of the key genes CD74, RPL26L1, and MRPL9 in the GSE19738 (MDD) dataset.

Using the eight core OSA-MDD genes, we further employed LASSO regression based on machine learning methods to identify diagnostic markers for OSA and MDD. LASSO regression with 10-fold cross-validation identified 0 potential OSA diagnostic marker (Figures 7A,B). For MDD diagnosis, LASSO regression identified 26 diagnostic markers (Figures 7C,D). In order to further narrow down and improve the screening of key genes, eight genes screened by four algorithms of cytoHubba were intersected with 26 key genes calculated by Lasso regression, and finally the intersection of disease-specific markers revealed three shared diagnostic markers for OSA with comorbid MDD: CD74 (CD74 molecule), RPL26L1 (ribosomal protein L26 like 1), and MRPL9 (mitochondrial ribosomal protein L9) (Figure 7E). Subsequently, receiver operating characteristic (ROC) analysis was performed to further evaluate the diagnostic potential of the three candidate hub genes. The results indicated significant diagnostic value for CD74, RPL26L1, and MRPL9 in discriminating both OSA (Figures 8A–C) and MDD (Figures 8D–F). To further validate these findings, an additional ROC analysis was conducted using an independent MDD dataset (GSE19738). The results confirmed the diagnostic utility of CD74 (Figure 8G) and RPL26L1 (Figure 8H) for MDD. However, MRPL9 did not perform above the random classifier threshold in the GSE19738 dataset (Figure 8I), supporting its exclusion from subsequent analyses.

### 3.7 Immune cell correlation analysis between hub genes and immune cells

**FIGURE 9:**
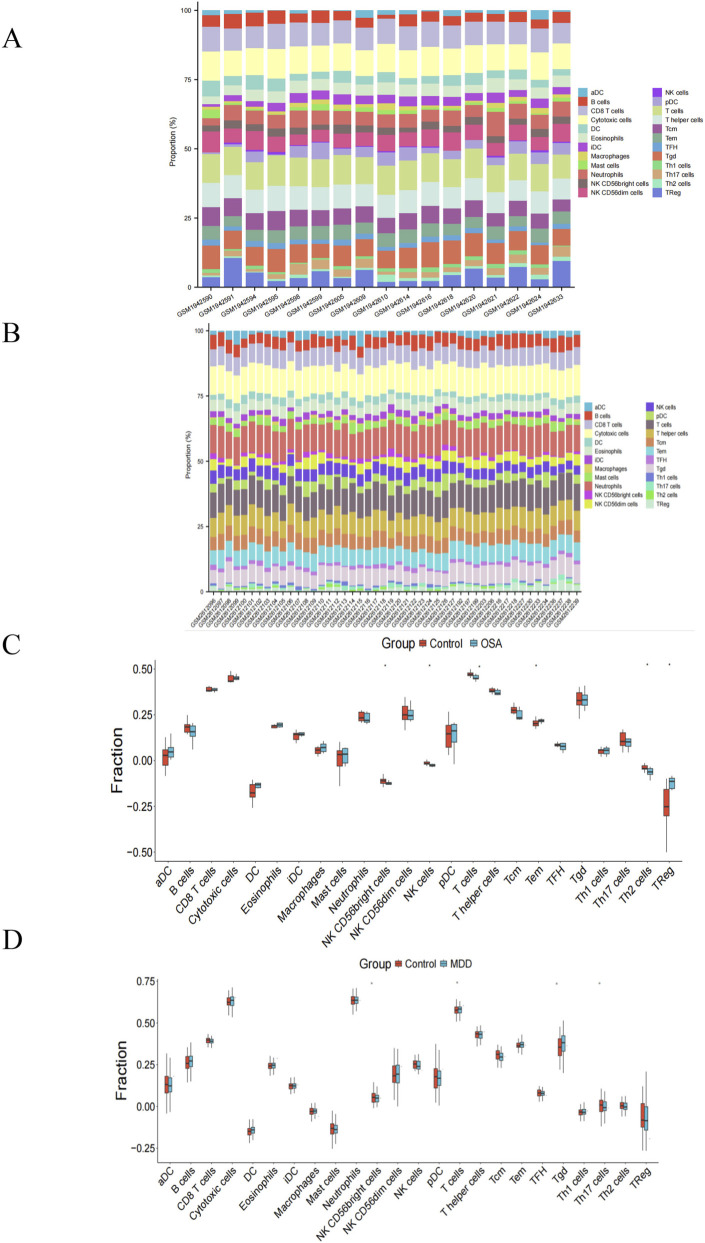
Immune cell correlation analysis in the OSA and MDD datasets. **(A,B)** Stacked bar charts of immune cell composition, showing the proportion of immune cell types in the OSA and MDD groups compared to the control group, respectively. **(C,D)** Distribution of immune cell types in the OSA and MDD groups, respectively. *P < 0.05; ***P < 0.001.

**FIGURE 10:**
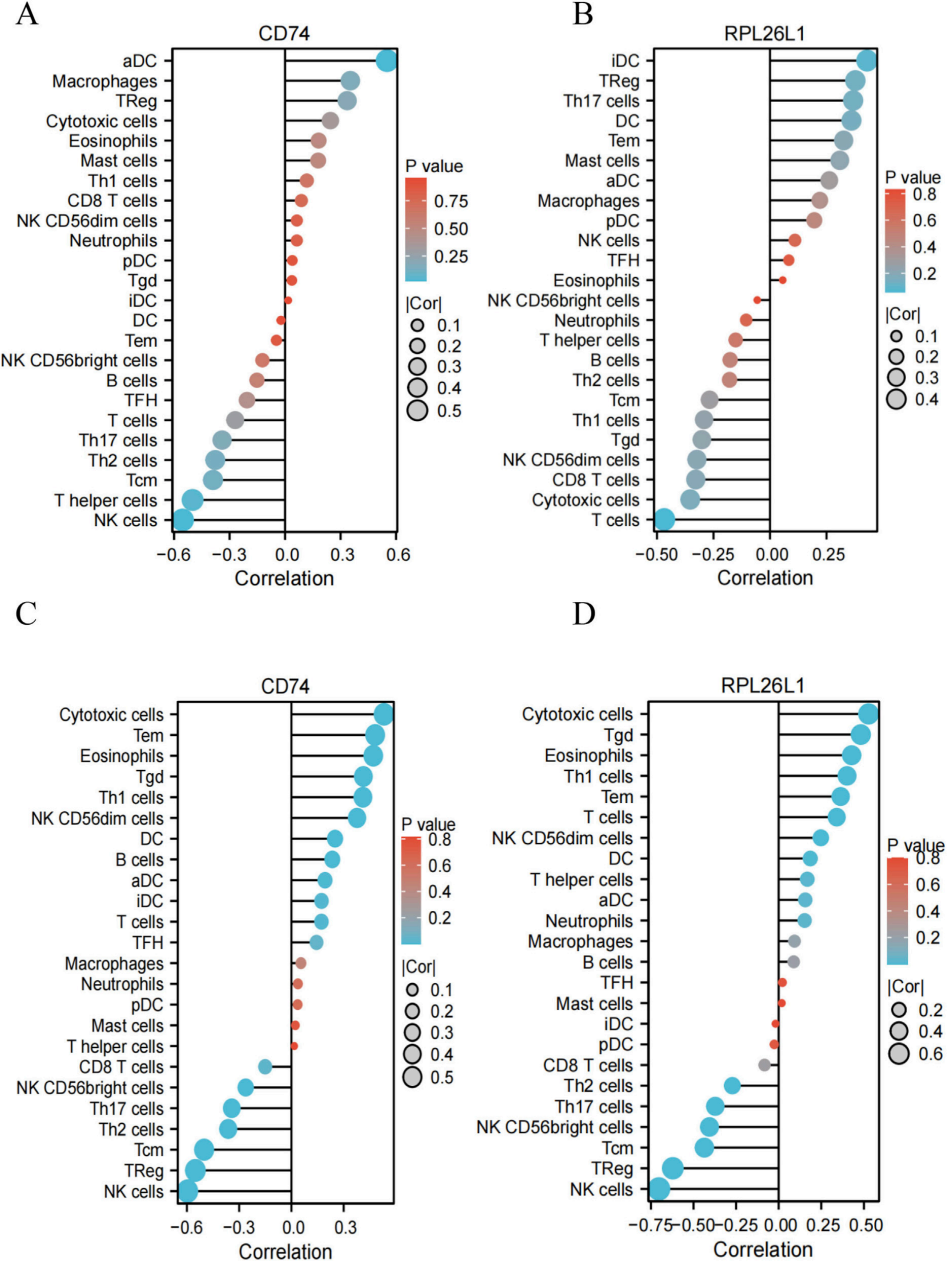
Spearman’s correlation analysis between hub genes and immune cells. **(A,B)** Correlation analysis between CD74 and RPL26L1 genes and immune cell types in the OSA disease group. **(C,D)** Correlation analysis between CD74 and RPL26L1 genes and immune cell types in the MDD disease group.

Considering that both OSA and MDD were closely related with infiltrating immune cells (Sarno et al., 2021; Ludwig et al., 2022; Poletti et al., 2024; Zhao et al., 2024), we performed ssGSEA analysis on the two hub genes, the results revealed significant differences in immune cell infiltration between patients and healthy controls. In OSA (Figures 9A,C), six immune cell types showed altered abundance: NK CD56bright cells, NK cells, T cells, and Th2 cells were significantly decreased in OSA patients compared to healthy controls, while Tem and Treg cells were elevated. For MDD (Figures 9B,D), four immune cell types differed significantly: NK CD56bright and Th17 cells were reduced in MDD patients, whereas T cells and γδ T cells were increased relative to controls. Besides, we found that the levels of T cells were significantly different in both OSA and MDD compared to healthy controls. Subsequently, Spearman’s correlation analysis revealed significant associations between the identified two hub genes (CD74 and RPL26L1) and immune cell infiltration levels in both OSA (Figures 10A,B) and MDD (Figures 10C,D).

### 3.8 *In vitro* validation of the hub genes using RT-PCR analysis

**FIGURE 11:**
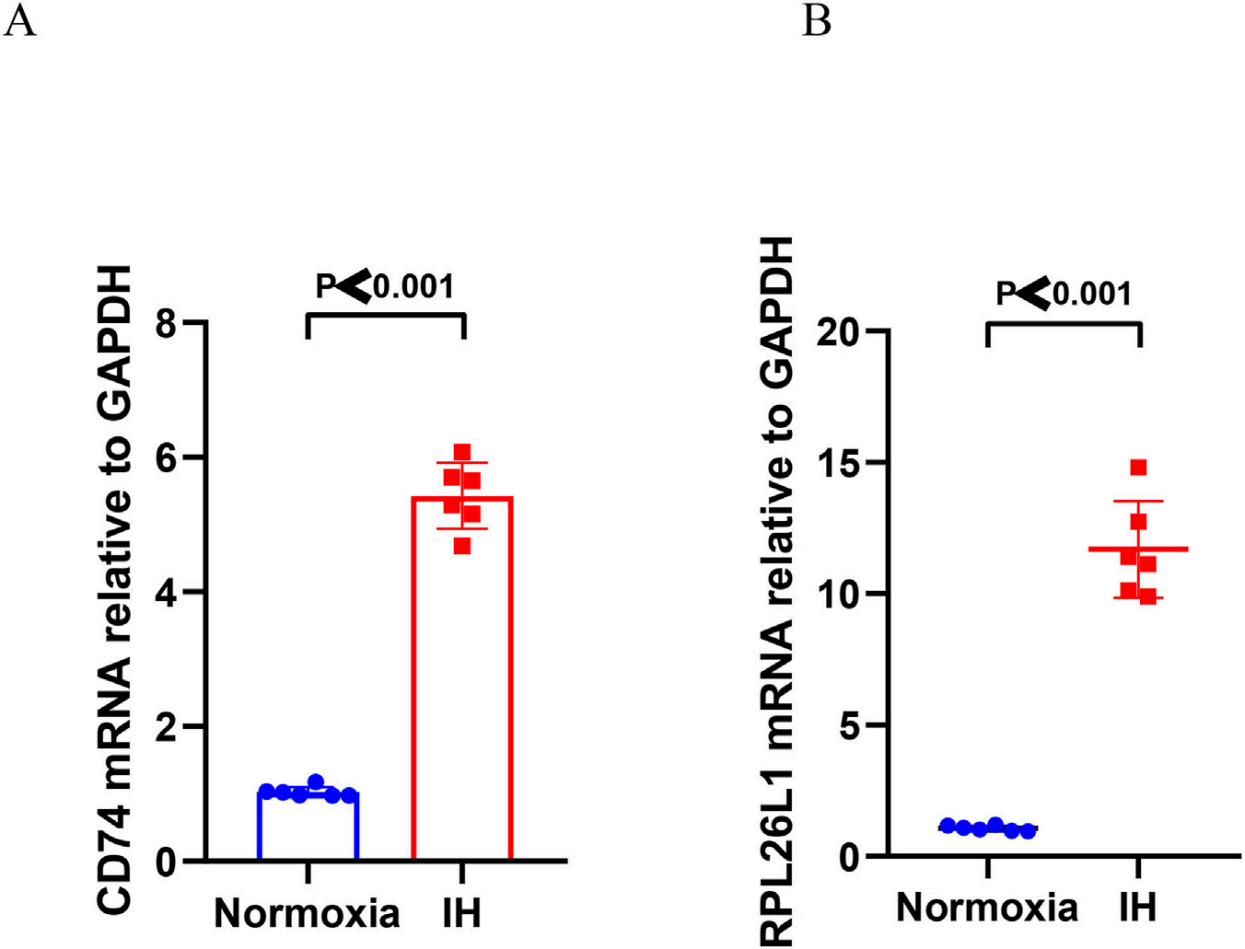
RT-PCR analysis of hub genes in the microglial cells. **(A)** The mRNA levels of CD74 in the IH microglial cells compared to that of normoxia microglial cells (∼5-fold upregulation). *P* < 0.001. **(B)** The mRNA levels of RPL26L1 in the IH microglial cells compared to that of normoxia microglial cells (∼10-fold upregulation). *P* < 0.001.

Subsequently, we performed *in vitro* experiments to further verify the expression of two hub genes, microglial cells exposed to IH for 48 h showed significantly increased expression of *CD74* (∼5-fold) (Figure 11A) and *RPL26L1* (∼10-fold) (Figure 11B) compared to normoxia controls, as measured by the RT-PCR analysis.

## 4 Discussion

The prevalence of the OSA in the general population is 9%–38% (Senaratna et al., 2017). MDD affects 6% of the general population, with OSA patients exhibiting MDD comorbidity rates of 0%–66% (Chinvararak et al., 2022). As the essential prevalent OSA comorbidity, depression remains frequently underdiagnosed and undertreated clinically. It significantly impacts OSA progression through multiple pathways: exacerbating disease activity, amplifying hypoxia-related comorbidities, reducing quality of life, and increasing mortality. Critically, OSA and depression establish a bidirectional pathological relationship that mutually intensifies symptoms. This interaction is partially explained by systemic inflammation and neuro-endocrine, and neuro-immune crosstalk mechanisms (Zhang et al., 2020). Patients with dual diagnoses require vigilant clinical monitoring to mitigate adverse outcomes.

Gene expression patterns elucidate mechanisms underlying OSA-MDD comorbidity and may reveal therapeutic targets. Using WGCNA analysis, we identified co-expression modules associated with both conditions. PPI analysis revealed eight OSA-associated hub genes linked to MDD. Functional enrichment analysis confirmed previously validated mechanisms (Briançon-Marjollet et al., 2014; Ye et al., 2022), such as leukocyte migration, leukocyte cell-cell adhesion, and aerobic respiration. ROC analysis demonstrated significant diagnostic value for most markers in classifying both OSA and MDD. ssGSEA analysis showed that the immune cell infiltration was significantly different between patients and controls in both OSA and MDD. Spearman’s correlation analysis revealed significant associations between the identified hub genes and immune cell infiltration levels in both OSA and MDD.

To further explore the diagnostic markers of OSA complicated by MDD, two diagnostic markers (CD74 and RPL26L1) were obtained from 34 core genes based on the two algorithms. *CD74* gene encodes a class II MHC-associated chaperone regulating antigen presentation and serves as a surface receptor for migration inhibitory factor (MIF). MIF binding activates CD74-mediated survival pathways and proliferation^31^. In OSA, hypoxia-inducible factor-1α (HIF-1α) upregulation in myocytes elevates MIF, which through CD74 interaction activates NF-κB, driving M1 macrophage polarization and inflammatory cytokine secretion (He et al., 2025). Clinically, elevated circulating MIF correlates with MDD progression and poor antidepressant response, with post-treatment normalization implicating the essential role of CD74 and MIF in MDD pathogenesis (Cristina Petralia et al., 2020). In our study, RT-PCR analysis confirmed that CD74 was increased in OSA patients compared to normal controls, WGCNA analysis confirmed a strong association between CD74 and OSA and MDD phenotypes, ROC analysis verified the diagnostic value of CD74 in both OSA and MDD conditions, ssGSEA analysis confirmed the association between infiltration of immune cells and the expression of CD74, collectively establishing its therapeutic potential for comorbid OSA-MDD.

The *RPL26L1* gene encodes a protein structurally homologous to ribosomal protein L26. Though currently understudied, RPL26L1 contributes to the pathogenesis of systemic lupus erythematosus, polycystic ovary syndrome, endometrial cancer, vitiligo, and mantle cell lymphoma through key pathways including RIG-I-like receptor signaling, antigen processing/presentation, p53 signaling, mitochondrial translation, and ribosomal assembly (Afshin Derakhshani et al., 2020; Yang et al., 2020; Guo et al., 2020; Hassan et al., 2022). Bioinformatics and biological evidence further implicate RPL26L1 as a pathological factor and therapeutic target in MDD (Feng et al., 2020). Notably, while RPL26L1 has not been previously associated with OSA, our multi-platform analysis demonstrates its significant upregulation in OSA patients (RT-PCR), strong phenotypic correlation with OSA and MDD (WGCNA analysis), diagnostic utility for both conditions (ROC curve), and linkage to immune cell infiltration (ssGSEA analysis), collectively establishing its therapeutic relevance for OSA-MDD comorbidity.

Evidence demonstrates that OSA-induced inflammation promotes leukocyte migration and adhesion (monocytes, macrophages, T cells, and so on) in both peripheral and vascular compartments (Briançon-Marjollet et al., 2014; Chuang et al., 2021). Concurrently, encephalitogenic Th17 cell infiltration and associated cytokines drive chronic neuroinflammation, perpetuating neurodegeneration in MDD (Shi et al., 2022). Besides, antigen presentation is uniquely tied to OSA-MDD interactions. CD74, a MHC class II chaperone, is upregulated by IH in microglia and mediates OSA’s HIF-1α/MIF signaling (He et al., 2025): this activates NF-κB and M1 macrophage polarization, amplifying systemic inflammation. In MDD, CD74 interacts with MIF to promote depressive-like behaviors (Cristina Petralia et al., 2020), and our ssGSEA data showed CD74 correlates with Treg/γδ T cell infiltration-immune subsets specific to OSA (Treg elevation) and MDD (γδ T cell increase). This CD74-immune axis directly bridges OSA’s hypoxia-driven immunity to MDD’s inflammatory pathophysiology. In our study, the key genes between OSA and MDD were mainly enriched in the leukocyte migration and leukocyte cell-cell adhesion pathways, suggesting that the antigen presentation and immunopathological mechanisms may underlie the OSA-MDD comorbidity.

Evidence demonstrates that OSA-induced IH activates microglia, generating oxidative stress that impairs aerobic respiration through mitochondrial damage, NADPH oxidase activation, and nitric oxide overproduction (Wu et al., 2021). This aligns with evidence linking altered mitochondrial dynamics to MDD pathophysiology. Critically, MDD patients exhibit compromised mitochondrial respiration-including reduced basal oxygen consumption, maximal electron transport system capacity, ATP-linked respiration, and spare respiratory capacity (Karabatsiakis et al., 2014; Boeck et al., 2018). Our enrichment identified hub genes in mitochondrial inner membrane and respiratory complexes-pathways directly disrupted by OSA-related IH. IH triggers mitochondrial ROS overproduction and ETC., impairment in microglia (Wu et al., 2021), while MDD patients exhibit reduced mitochondrial respiration (e.g., ATP-linked oxygen consumption (Karabatsiakis et al., 2014)). Critically, our *in vitro* data showed IH upregulates hub genes, this upregulation likely reflects a compensatory response to IH-induced ETC., damage, which in turn exacerbates MDD-related neuronal energy deficit-creating a bidirectional OSA-MDD loop. Besides, OSA’s IH directly inhibits OXPHOS by destabilizing, ETC., complexes (Wu et al., 2021), while MDD is associated with OXPHOS gene downregulation (Boeck et al., 2018). Our hub genes are essential for OXPHOS: their dysregulation in IH reduces ATP production and increases ROS, activating microglia to secrete pro-inflammatory cytokines (e.g., IL-6, TNF-α). These cytokines, in turn, perpetuate MDD’s neuroinflammatory state (Gupta and Simpson, 2015)-specifically connecting OSA’s metabolic stress to MDD’s inflammatory pathogenesis.

Both OSA and MDD patients exhibit dysregulated innate and adaptive immunity. OSA demonstrates altered monocyte distribution-decreased classical (CD14^++^CD16^−^) but elevated intermediate (CD14^+^CD16^+^) and non-classical (CD14^dim+^CD16^+^) subsets versus healthy controls-alongside increased peripheral TH17 cells and TH17/Treg ratios correlating with disease severity (Ye et al., 2012; Ludwig et al., 2022). MDD shows strong comorbidity with inflammatory and autoimmune conditions, particularly inflammatory bowel disorder (Whitehouse et al., 2019), and elevated depression rates in type 1 diabetes (15.2%), rheumatoid arthritis (15%), multiple sclerosis (18%), and Guillain-Barré syndrome (6.7%) (Sarno et al., 2021). Critically, our identified OSA-MDD hub genes (CD74 and RPL26L1) correlate significantly with immune cell infiltration, while differential infiltration patterns between patients and controls implicate immune-mediated mechanisms in OSA-MDD shared pathogenesis.

While similar bioinformatics approaches exist for other MDD-related comorbidities (Zhou TT et al., 2023; Hu et al., 2024), our study advances prior work in three key ways specific to OSA-MDD: 1) OSA-specific mechanistic validation: We linked hub genes to IH-the core driver of OSA-via *in vitro* experiments showing their upregulation in IH-exposed microglia, a step absent in prior comorbidity studies. 2) Tailored immune infiltration analysis: We identified OSA-MDD-specific immune perturbations (e.g., shared T cell dysregulation, OSA-unique Tem/Treg elevation, and MDD-unique γδ T cell increase) and correlated them with diagnostic markers, providing gene-immune links not reported elsewhere. 3) Focus on mitochondrial OXPHOS and antigen presentation pathways: Our enrichment and validation studies highlighted mitochondrial dysfunction as a convergent OSA-MDD mechanism-an understudied pathway in prior comorbidity research.

Our study acknowledges several limitations. Firstly, relatively small OSA and MDD cohort sizes warranting future validation with larger datasets. Secondly, insufficient mechanistic exploration linking immune cell infiltration to hub genes (CD74/RPL26L1) in disease pathogenesis. Thirdly, absence of additional molecular biology validation (e.g., MDD model validation). Fourthly, lack of computational analyses (e.g., molecular docking and molecular dynamic simulation) to characterize binding affinities. Fifthly, there is a lack of additional OSA and MDD datasets to verify the diagnostic value of hub genes.

## 5 Conclusion

The comorbidity of OSA and MDD is driven by shared dysregulation in mitochondrial OXPHOS and antigen presentation pathways, mediated by key hub genes CD74 and RPL26L1, which promote neuroinflammation and disrupt cellular energy metabolism via altered immune cell infiltration. We hypothesize that CD74 and RPL26L1 serve as dual diagnostic biomarkers and functionally contribute to disease pathophysiology, making them compelling candidates for therapeutic targeting in OSA-MDD comorbidity.

## Data Availability

The article contained the data that could be accessed in the public domain through the NCBI GEO database, including the GSE75097, GSE98793, and GSE19738 datasets.
