# Supplementary material for: Potential diagnostic markers and therapeutic targets for obstructive sleep apnea with comorbid depression based on bioinformatics analysis
Source: Front Genet. 2025 Nov 19;16:1655000. doi: 10.3389/fgene.2025.1655000 (PMC12673486; doi:10.3389/fgene.2025.1655000)
Supplement: Supplementary file 1 [file DataSheet1.docx]

**Supplementary Materials**

**
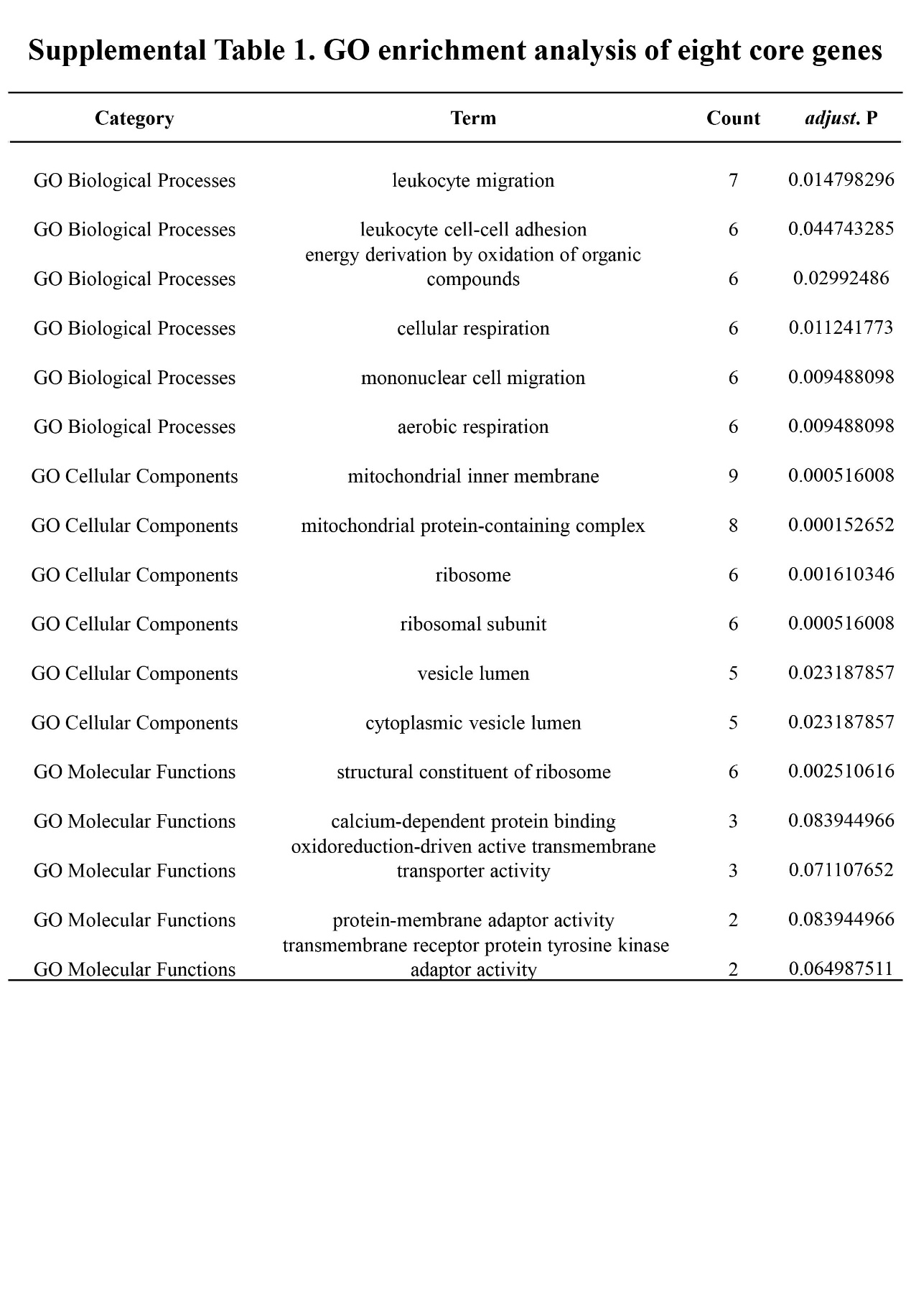
**

**
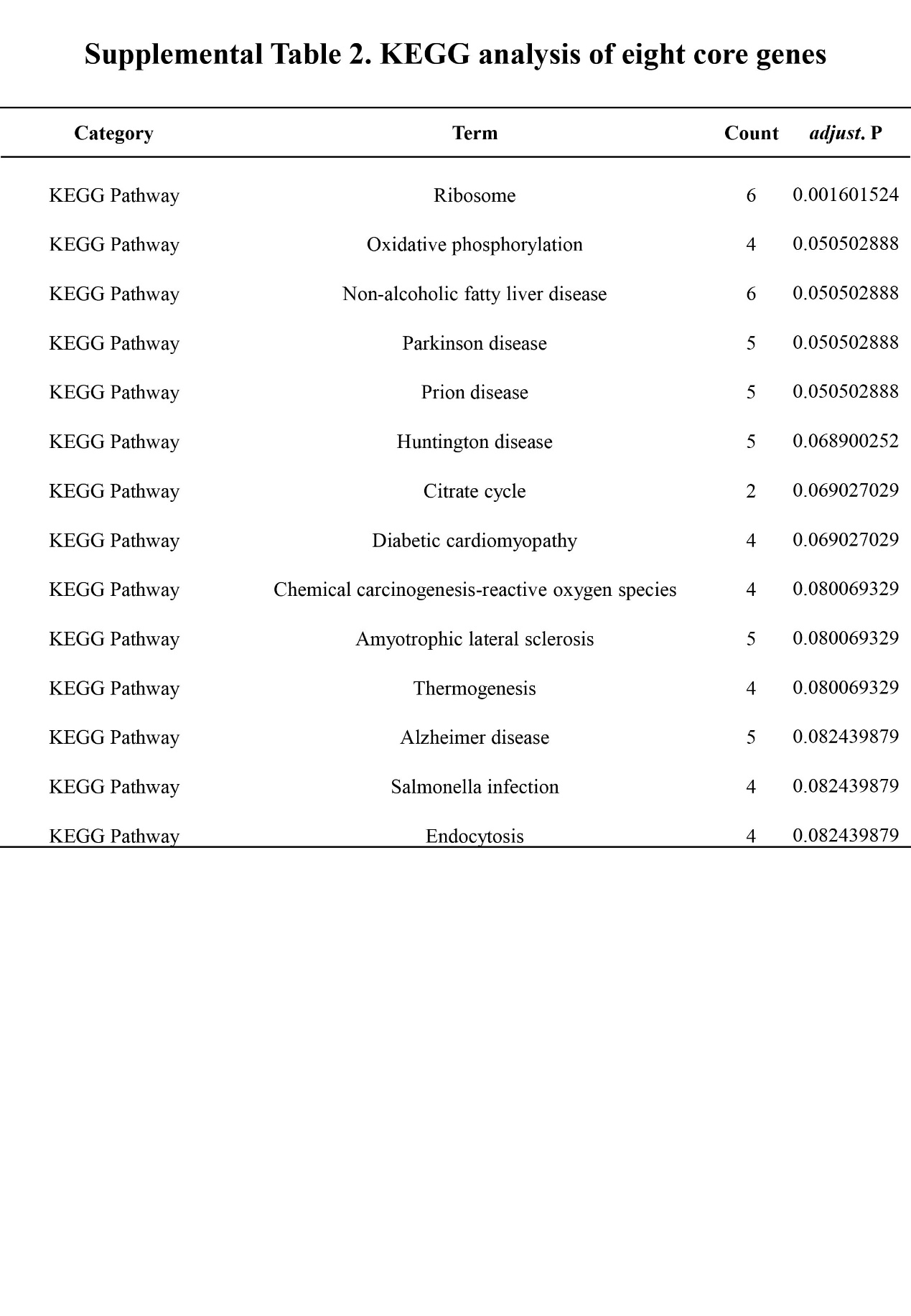
**

**Supplemental Figure 1**

**
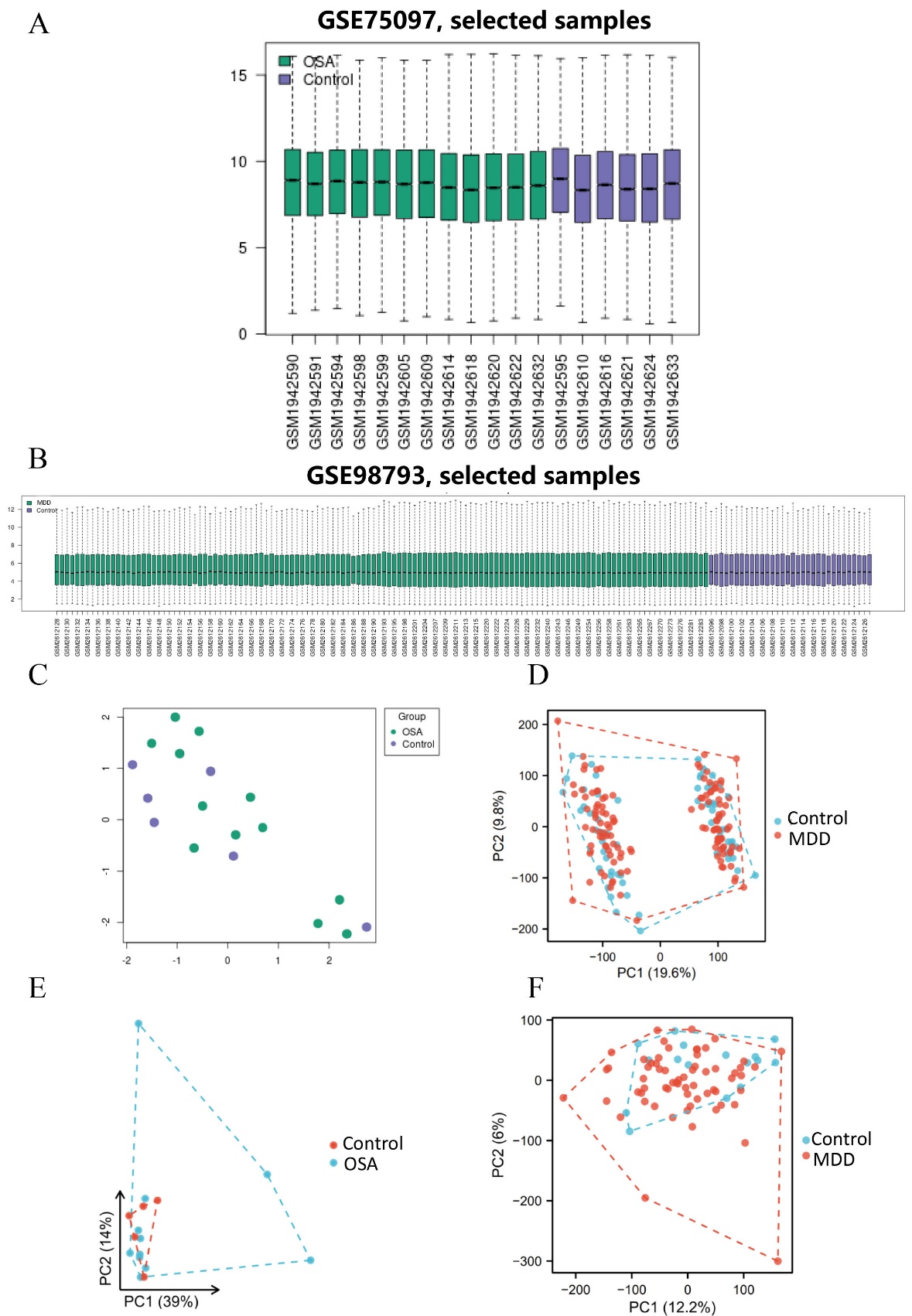
**

**Supplemental Figure 1. The expression matrices of datasets.** (A,B) The expression matrices of GSE75097 (A) and GSE98793 (B) datasets. (C,D) The PCA analysis of GSE75097 (C) and GSE98793 (D) datasets before ComBat correction. (E,F) The PCA analysis GSE75097 (E) and GSE98793 (F) datasets after ComBat correction.

**Supplemental Figure 2**

**
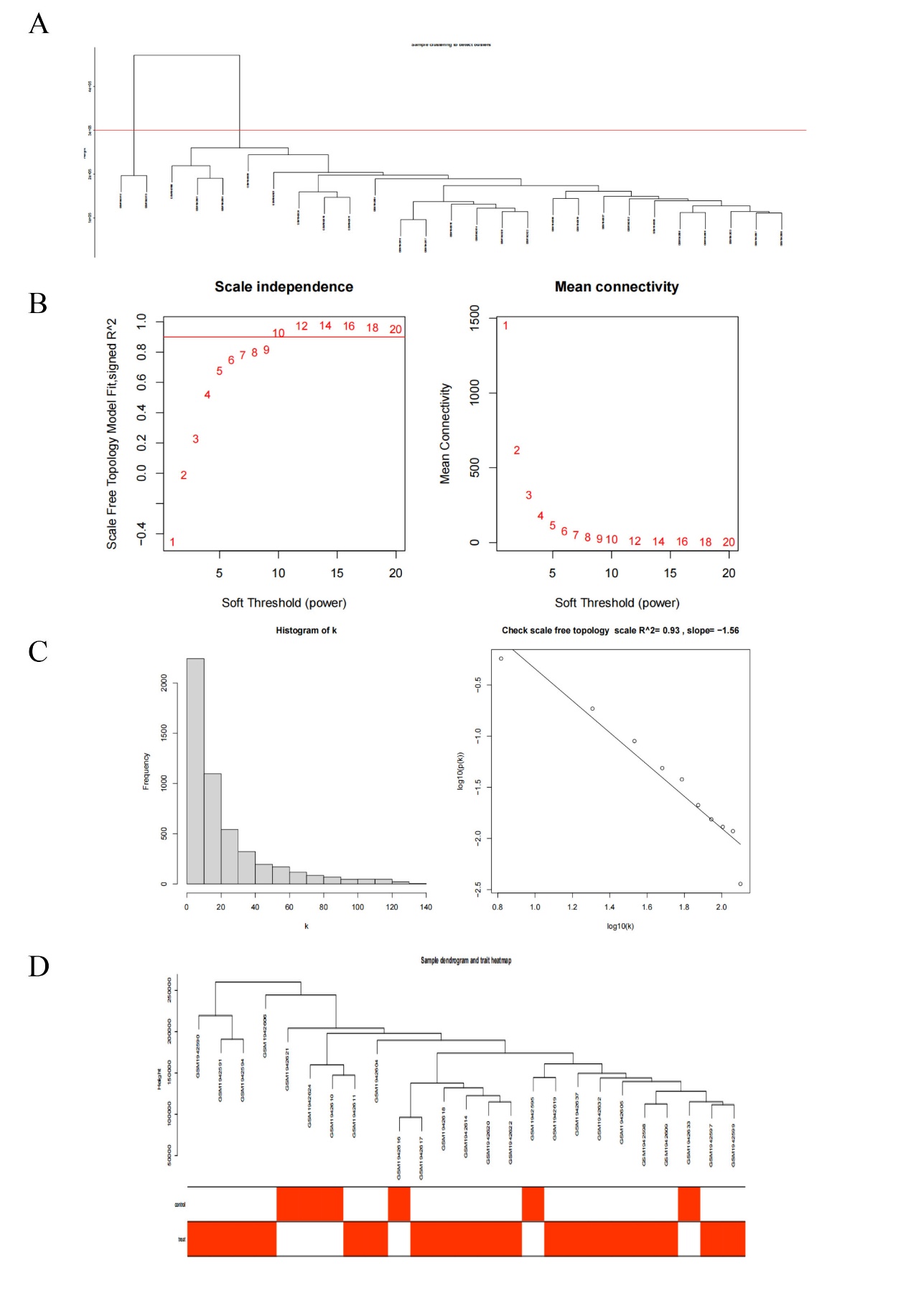
**

**Supplemental Figure 2. Hierarchical clustering, scale independence, and module division of the** **GSE75097 dataset.** (A) The hierarchical clustering trees showing the genes from the GSE75097 dataset. (B) The scale independence and mean connectivity were calculated to select an appropriate soft threshold (power). The mean connectivity plot showed the average number of connections between genes at different soft thresholds. (C) The histogram displaying the gene degree distribution and the check of topological scale. (D) The hierarchical clustering trees showing the division of gene modules.

**Supplemental Figure 3**


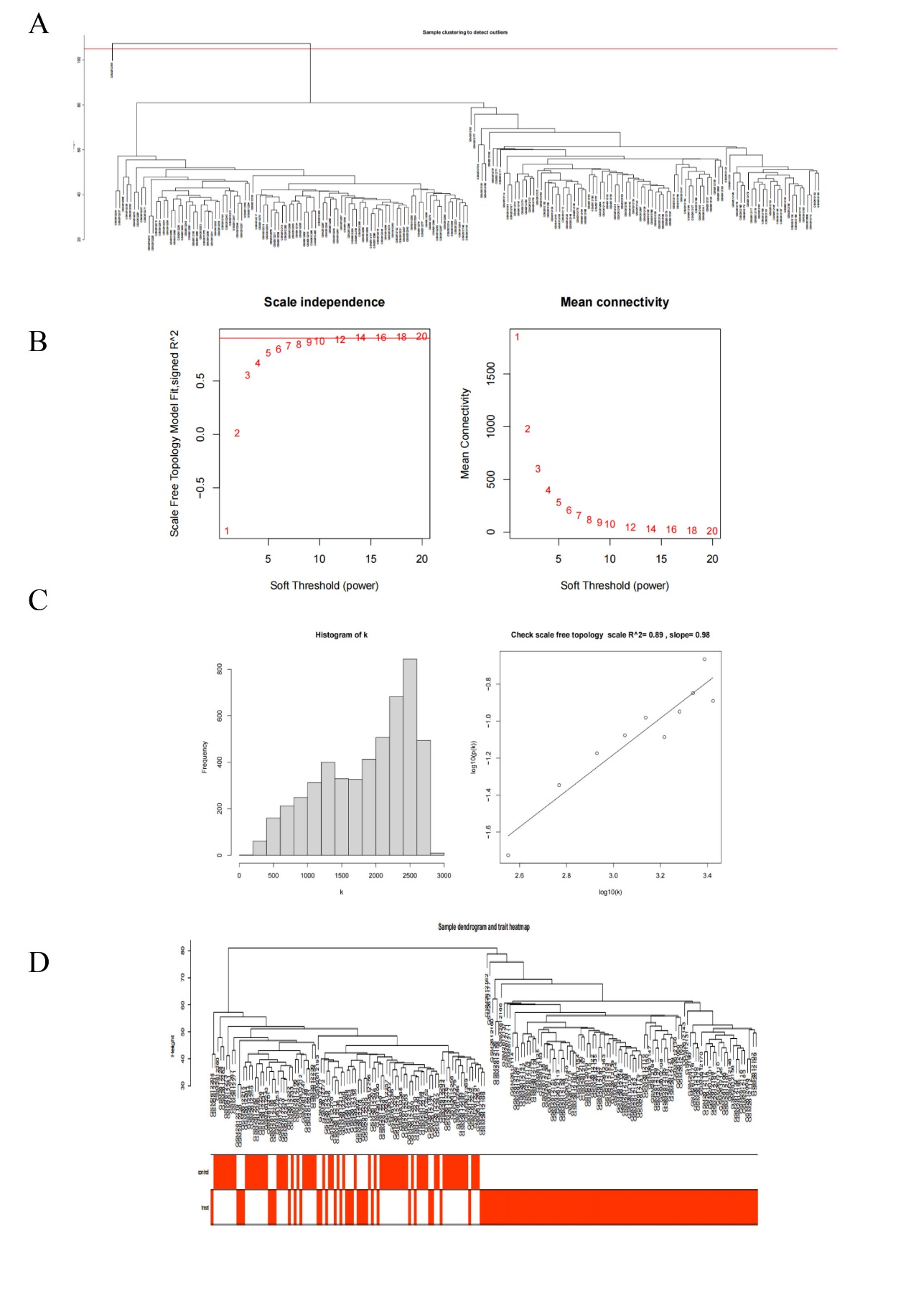


**Supplemental Figure 3.** **Hierarchical clustering, scale independence, and module division of the** **GSE98793 dataset.** (A) The hierarchical clustering trees showing the genes from the GSE98793 dataset. (B) The scale independence and mean connectivity were calculated to select an appropriate soft threshold (power). The mean connectivity plot showed the average number of connections between genes at different soft thresholds. (C) The histogram displaying the gene degree distribution and the check of topological scale. (D) The hierarchical clustering trees showing the division of gene modules.
